# Supplementary material for: The Dynamics of Cryptococcus neoformans infection in Galleria mellonella
Source: bioRxiv. 2025 Mar 20:2025.03.19.644221. Originally published 2025 Mar 19. Preprint. [Version 2] doi: 10.1101/2025.03.19.644221 (PMC11957108; doi:10.1101/2025.03.19.644221)
Supplement: 4 [file NIHPP2025.03.19.644221v2-supplement-4.pdf]

bioRxiv preprint doi: https://doi.org/10.1101/2025.03.19.644221; this version posted March 20, 2025. The copyright holder for this preprint (which was not certified by peer review) is the author/funder, who has granted bioRxiv a license to display the preprint in perpetuity. It is made available under aCC-BY 4.0 International license.

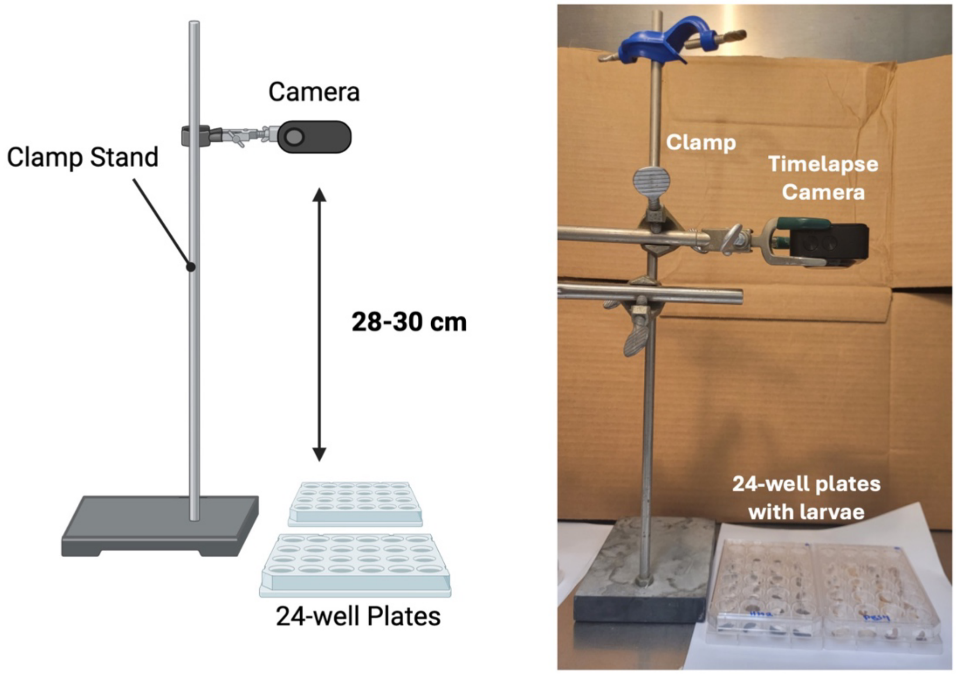

Supplementary Figure 1. Timelapse photography set up. Timelapse camera is suspended above two 24-well plates containing *G. mellonella* larvae using a 3-pronged clamp attached to a support base.
